# Unmasking MRSA’s Armor: Molecular Mechanisms of Resistance and Pioneering Therapeutic Countermeasures

**DOI:** 10.3390/microorganisms13081928

**Published:** 2025-08-18

**Authors:** Yichen Liu, Hao Lu, Gaowei Hu, Jiaqi Liu, Siqi Lian, Shengmei Pang, Guoqiang Zhu, Xueyan Ding

**Affiliations:** 1 College of Veterinary Medicine, Henan Agricultural University, Zhengzhou 450046, China; 13137008063@163.com; 2 Molecular Biology Laboratory, Zhengzhou Normal University, Zhengzhou 450044, China; luhao79@outlook.com; 3 College of Life Sciences, Taizhou University, Taizhou 318000, China; hugaowei68@163.com; 4 College of Veterinary Medicine, Yangzhou University, Yangzhou 225009, China; dx120220189@stu.yzu.edu.cn (J.L.); mx120180715@yzu.edu.cn (S.L.); shengmeipang@163.com (S.P.); yzgqzhu@yzu.edu.cn (G.Z.); 5 Henan Province Key Laboratory of Animal Food Pathogens Surveillance, Zhengzhou 450046, China; 6 Ministry of Education Key Laboratory for Animal Pathogens and Biosafety, Zhengzhou 450046, China

**Keywords:** MRSA, drug-resistance mechanisms, novel treatment strategies, public health safety

## Abstract

Methicillin-resistant *Staphylococcus aureus* (MRSA), characterized by high-level β-lactam resistance and increasing multi-drug resistance, poses a severe and growing global threat to human health and public safety. This review examines MRSA’s complex resistance mechanisms, including *mecA*/*mecC*-mediated expression of low-affinity PBP2a, regulatory roles of auxiliary genes like *fem* and *vanA*, enzymatic inactivation by β-lactamases and modifying enzymes, efflux pump activity, and biofilm formation. We also systematically review novel therapeutic strategies, such as combination therapies, phage-derived biofilm disruptors, membrane-targeting silver nanoparticles, cell-penetrating antimicrobial peptides, colonization-competitive probiotics, and antibiotic-synergizing phytochemicals. These advances provide critical insights for developing effective countermeasures against MRSA, while highlighting the urgent need for global collaboration, antibiotic stewardship, and innovative drug development to combat antimicrobial resistance.

## 1. Introduction

*Staphylococcus aureus* is a common Gram-positive pathogen that represents a significant cause of both healthcare-associated and community-acquired infections. With the development and application of antibiotic drugs, the death rate of *Staphylococcus aureus* infection has decreased significantly, but more and more drug-resistant strains have emerged. Methicillin-resistant *Staphylococcus aureus* (MRSA) was isolated for the first time in 1961 after methicillin was used to effectively control the infection of penicillin-resistant *Staphylococcus aureus* in 1959. Inappropriate use of antimicrobials has led to a growing epidemic of MRSA. MRSA showed extensive resistance to β-lactam antibiotics and simultaneous resistance to aminoglycosides, fluoroquinolones, tetracycline, macrolides, lincomycin, and other antibiotics [1]. This multiple and complex resistance mechanism significantly increases the fatality rate of MRSA infection and may change over time, which brings great challenges to clinical treatment and endangers the public health safety of humans and animals. Therefore, it has become urgent to continuously monitor the resistance pattern of MRSA and develop new treatment strategies to deal with MRSA infection, which is of great significance for clinical guidance and treatment.

The resistance mechanism of MRSA is multiple and complex, among which the resistance to methicillins is mainly attributed to the *mecA* gene carried by MRSA, which can encode a PBP2a protein that is insensitive to all β-lactam antibiotics (such as penicillin, cephalosporins and carbapenems) and thus resist the action of such antibiotics [2]. MRSA can not only activate the inherent drug-resistance mechanism to produce drug resistance by changing the target of antibacterial drugs and utilizing the efflux pump encoded by chromosomes, but also activate the acquired drug-resistance mechanism through gene mutation and acquisition of exogenous drug-resistance genes, thereby affecting the sensitivity of bacteria to antibiotics [3]. In addition, MRSA can also form a complex drug-resistance mechanism by forming biofilms and changing the affinity of antimicrobial targets. In this review, the mechanism of MRSA resistance caused by different factors and new therapeutic strategies to avoid more severe MRSA resistance were reviewed, and the future research direction was prospected, aiming at providing new ideas for the subsequent prevention and control of MRSA.

## 2. Drug-Resistance Mechanisms of MRSA

**Figure 1:**
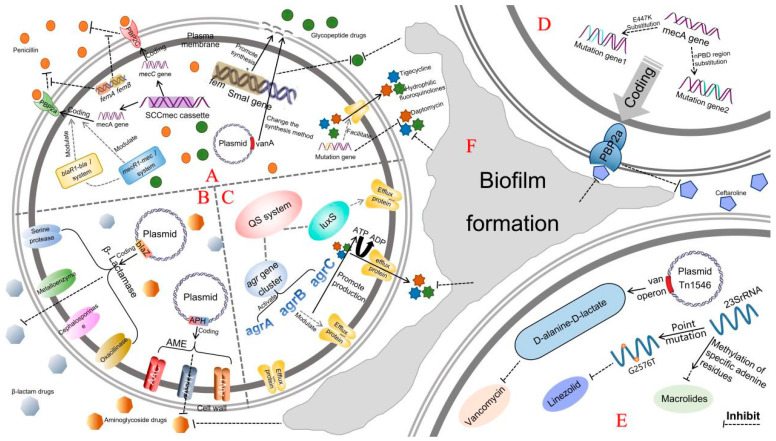
A comprehensive overview of the resistance mechanisms of MRSA. (**A**) Gene-mediated resistance: The *mec* genes (*mecA*/*mecC*) encode low-affinity penicillin-binding proteins (PBP2a/PBP2c), conferring intrinsic resistance to all β-lactam antibiotics (e.g., methicillin, penicillin, cephalosporins). Concurrently, *fem* genes enhance cell wall integrity by regulating peptidoglycan biosynthesis, synergizing with the plasmid-acquired *vanA* gene to drive heteroresistance. This *vanA*-encoded enzyme synthesizes D-alanine-D-lactate to modify cell wall precursors, establishing glycopeptide resistance. Critically, the SCCmec chromosomal “resistance island” harbors both *mecA* and multi-drug-resistance determinants, propagating resistance to β-lactams, aminoglycosides, and other antibiotic classes through horizontal genetic transfer. (**B**) β-lactamases and aminoglycoside modifying enzyme (AME)-mediated resistance: β-lactamases encoded by the *blaZ* gene of the plasmid include serine proteases, metalloenzymes, cephalosporins, and oxacillinases, which inactivate β-lactam antibiotics by hydrolyzing the β-lactam ring. Similarly, the aminoglycoside-modifying enzyme AME encoded by the plasmid APH gene, containing acetyltransferase (AAC), phosphotransferase (APH), and nucleoside transferase (ANT), chemically modifies aminoglycoside drugs to reduce their binding to ribosomes. (**C**) Efflux pump-mediated drug resistance: QS system can deactivate agrA-agrB-agrC operons through *agr* gene, generate AI-2 through luxS pathway to synergistically amplify agr signal, and then the activated efflux pumps pump fluoroquinolones, macrolides, tetracycline, and other drugs out of the cell by ATP or proton gradient. (**D**) Gene-mutation-mediated drug resistance: substitution mutations in key sites (such as E447K) or penicillin-binding domain (PBD) of the *mecA* gene can produce mutant genes, resulting in reduced affinity of the encoded PBP2a protein for specific β-lactam antibiotics such as ceftaroline, resulting in drug resistance. (**E**) Drug resistance mediated by target alteration: The van operon carried by plasmid Tn1546 can synthesize D-Ala-D-Lac peptidoglycan precursor, thereby inhibiting the binding of vancomycin, while the point mutation G2576T in 23S rRNA weakens the affinity of linezolid, and the methylation of its specific adenine residue also leads to macrolide resistance. (**F**) Biomembrane-mediated drug resistance: The state of the biomembrane can significantly enhance the tolerance of bacteria. The *mecA* gene and the QS/agr system are crucial for the structure and resistance of the biomembrane. Mutations in its related genes and methylation at the G2576T/A site of 23S rRNA are also associated with the resistance to β-lactam, macrolide, and aminoglycoside antibiotics.

The drug-resistance mechanisms of MRSA are complex and diverse, involving multiple aspects such as gene-mediated resistance, enzyme production, drug efflux pumps, biofilm formation, and alterations in target sites (Figure 1). Central to β-lactam resistance is the *mecA* gene, which encodes the low-affinity penicillin-binding protein 2a (PBP2a). Unlike constitutively expressed PBPs that exhibit high binding affinity for β-lactams, PBP2a remains unaffected by these antibiotics due to its markedly reduced drug binding capacity. When β-lactams inactivate endogenous PBPs, PBP2a compensates by performing the critical transpeptidase function required for cell wall biosynthesis, thereby enabling bacterial survival under antimicrobial pressure [4]. This core resistance mechanism is further potentiated by auxiliary genes (e.g., *fem*, *vanA*) through synergistic regulation [5,6]. Concurrently, MRSA employs β-lactamases and antibiotic-modifying enzymes to directly inactivate or structurally alter antibiotics [7], while target site modifications reduce drug-target binding affinity [8]. These evasion strategies are complemented by efflux pumps that diminish intracellular antibiotic accumulation [9] and biofilm formation that provides physical protection in hostile environments [10]. Collectively, these multilayered resistance mechanisms pose significant therapeutic challenges, necessitating urgent development of novel strategies to counter this critical public health threat.

### 2.1. Gene-Mediated Drug-Resistance Mechanisms

#### 2.1.1. *mec* Gene-Mediated Drug Resistance

In the MRSA gene sequence, the *mec* gene is the main determinant of MRSA drug resistance. The *mec* gene is an exogenous DNA insertion on the bacterial chromosome, about 30 to 50 kp in size, usually located near the pur-nov-his gene group. In MRSA, the chromosomally mediated inherent resistance mechanism is mainly due to the acquisition of a new gene encoding penicillin-binding proteins (PBPs), *mecA*, which can be integrated into methicillin-sensitive Staphylococcal chromosome cassette mec (SCCmec). The *mecA* gene encodes the expression of penicillin-binding protein 2a (PBP2a), a PBP with low affinity for β-lactam antibiotics, including methicillin, and thus little or no binding by β-lactam antibiotics, showing inherent resistance [11,12]. PBP2a is a class of DD-transeptidases with a unique function, which also has the ability to participate in bacterial cell wall synthesis with the inherent PBPs of *Staphylococcus aureus*, but it is exactly the opposite of the high affinity of PBPs for β-lacamases antibiotics. Beta-lactam antibiotics inhibit the synthesis of bacterial cell walls by irreversibly acylating serine residues in bacterial PBPs and inactivating them. In contrast, PBP2a produced by MRSA can bypass this inhibition by not being acylated by beta-lactam antibiotics and still catalyze the critical DD transpeptide reaction required by the cell wall [13]. Therefore, when β-lactam antibiotics bind to PBPs and cause them to be inactivated, PBP2a can replace these inactivated PBPs to perform the function of transeptides, maintain cell wall synthesis, and enable bacteria to survive and resist the killing effect of β-lactam drugs [14].

The *mecA* gene is a specific resistance gene of MRSA and plays a decisive role in its resistance. The transcriptional expression of *mecA* is controlled by two regulatory systems, namely, the *mecA* regulatory system (*mecR1*-*mecI* system) and the β-lactamase regulatory system (*blaR1*-*blaI* system) [15]. These two regulatory systems are functionally similar, and they participate in the regulation of the *mecA* gene through their own mechanisms. Although both systems can affect *mecA* gene expression, the *mecR1*-*mecI* system is generally superior to the *blaR1*-*blaI* system in terms of regulatory intensity [16,17]. Resistance in most MRSA strains is primarily due to the presence and expression of the *mecA* gene, although there are a small number of MRSA strains that do not carry the *mecA* gene and rely instead on other resistance mechanisms. Statistics show that more than 90% of MRSA strains carry the *mecA* gene, and the presence of *mecA* genes is almost universal in strains that are highly resistant to beta-lactam antibiotics. Therefore, the *mecA* gene plays a central role in MRSA strains and is a major factor in their inherent drug-resistance mechanism [4].

In addition to the *mecA* gene mentioned above, the MRSA SCCmec element has been shown to have a homologous gene of the *mecA* gene, namely the *mecC* gene. The *mecC* gene can encode the PBP2c protein, whose function is similar to the PBP2a protein, which can make MRSA resistant to β-lactam antibiotics [18]. The discovery of the *mecC* gene suggests that the MRSA resistance mechanism may be more complex than previously thought, and that there are multiple pathways that can lead to bacterial resistance to beta-lactam antibiotics.

#### 2.1.2. *fem* Gene-Mediated Drug Resistance

Although the *mecA* gene is the key to MRSA resistance to methicillin, the expression level of PBP2a has no direct strong correlation with its resistance level, and auxiliary genes such as the *fem* gene (also known as *aux* gene) are also involved in drug resistance. These cofactors are dispersed throughout the bacterial chromosomes and synergically regulate cell wall synthesis to enhance drug resistance. At present, a variety of *fem* genes have been discovered, among which *femA*, *femB*, *femC*, *femD*, *femE*, and *femF* genes have been relatively studied. These genes regulate the expression level of PBP2a protein by directly or indirectly participating in the biosynthesis process of the bacterial cell wall, thus affecting the resistance of MRSA to methicillin [5,19,20]. The insertion inactivation of the *femA* and *femB* genes, which are located on the *SmaI-A* fragment, results in the complete loss of MRSA resistance to methicillin, but this does not affect the production of the PBP2a protein. This suggests that *femA* and *femB* may regulate the activity or function of PBP2a through other mechanisms that influence MRSA’s sensitivity to methicillin. *femC* and *femD* genes play a complex role in regulating MRSA resistance and may be involved in multiple biological processes at the same time. Both are located on the *SmaI-A* and *SmaI-I* fragments, and their insertion inactivation results in a decrease in the basal resistance level of MRSA, but at the same time may lead to the formation of highly resistant subclones [21]. The insertion inactivation of the *femE* gene, which is located on the *SmaI-A* fragment, does not appear to have a significant effect on the level of basal MRSA resistance. This suggests that *femE* may not be the primary acting factor in regulating MRSA resistance, or that its mechanism of action is unclear. The *femF* gene is located on the *SmaI-B* fragment, and its insertion inactivation can lead to heterogeneous bacterial expression resistance. These results suggest that *femF* may affect the gene expression pattern of MRSA and lead to the heterogeneous expression of drug resistance.

#### 2.1.3. *vanA* Gene-Mediated Drug Resistance

The transfer of the *vanA* gene is closely related to the increase of MRSA resistance to glycopeptide antibiotics. Tn1546 is a transposon containing the *vanA* gene, which was originally present in *Enterococcus*, but can pass the *vanA* gene to *Staphylococcus aureus* through plasmid-mediated horizontal gene transfer. This gene transfer event caused *Staphylococcus aureus* to acquire resistance to glycopeptide antimicrobials, because the protein encoded by the *vanA* gene changes the way the bacterial cell wall is synthesized, making it difficult for glycopeptide antimicrobials to bind to the bacterial cell wall, thus losing their bactericide effect [6].

The emergence of VISA and VRSA exacerbates the difficulty of clinical treatment, as these strains are more resistant to antibiotics and treatment options are limited. The transfer of the *vanA* gene and its expression in *Staphylococcus aureus* is one of the important mechanisms leading to the resistance of MRSA to glycopeptides. This finding has important implications for guiding clinical treatment and preventing the spread of drug-resistant strains.

#### 2.1.4. SCCmec-Mediated Drug Resistance

SCCmec is the site of drug-resistance gene insertion and accumulation, also known as the “drug resistance island” of *Staphylococcus aureus*, where almost all drug-resistance genes are located. The element is mobile and can be transferred between the chromosomes of *Staphylococcus aureus* [22]. MRSA SCCmec has acquired the gene encoding methicillin resistance, and its self-cutting and recombination capabilities, as well as the function of resistance island, make it an important carrier for the transmission of resistance genes, and the fundamental reason for the continuous expansion of the resistance spectrum [22,23]. In addition, the element also carries multiple antimicrobial resistance genes, including but not limited to *mecA*, resulting in multiple resistance to MRSA [24].

#### 2.1.5. Genetic Mutation-Mediated Drug Resistance

In addition to the core drug-resistance mechanism mainly mediated by the *mec* gene and assisted by the *fem* and *vanA* genes mentioned above, gene mutation is also another key factor in the activation of MRSA acquired resistance. The high resistance of MRSA to hydrophilic fluoroquinolines is due to the mutation of *grlA* and *gyrA* genes in bacteria, and the increased expression of the *norA* gene enables the multi-drug efflux pump NorA to quickly pump drugs out of bacteria [25]. In the study of MRSA resistance to tigecycline (tetracycline derived glycycline), Dabul et al. found that mutations in transcriptional regulator *mepR* and efflux pump *mepA* led to increased efflux of drugs, making them resistant to tigecycline [26]. It was also found that MRSA strains resistant to daptomycin showed changes in cell membrane characteristics, such as enhanced membrane fluidity, increased surface positive charge, decreased sensitivity to depolarization reaction, weakened binding force with daptomycin, etc. [27]. These changes may be related to *mprF* gene mutations. The bifunctional membrane protein encoded by the *mprF* gene is responsible for the synthesis and transport of lysyl phosphatidylglycerol, and the gene mutation leads to the increase of the lysyl phosphatidylglycerol content on the outer surface of the cell membrane, increasing the positive charge, and thus reducing the sensitivity of MRSA to daptomycin.

Although most research on drug-resistant gene mutations is still lacking specific data, with the increase in drug-resistant strains, the exploration of progress in this area is urgent. In addition, with the abuse of antibiotics, the multiple complex drug-resistance mechanisms caused by the combination of mutated new genes and inherent drug-resistance genes have become a major difficulty in clinical breakthroughs, and the research on MRSA-related gene mutations has become a focus of attention.

### 2.2. Production of Inactivated and Modified Enzymes

#### 2.2.1. β-Lactamases

In addition to the intrinsic resistance mediated by chromosome DNA, another equally important acquired resistance mechanism of MRSA is related to β-lactamase produced by plasmid mediated DNA insertion, transduction, and transformation. β-lactamases, as a class of important inducer enzymes, are mainly used to catalyze the hydrolysis of β-lactam bonds in β-lactam antibiotic molecules, which leads to the inactivation of antibiotics and leads to bacterial resistance. MRSA achieves β-lactamase expression by acquiring β-lactamase coding genes (usually from mobile genetic elements such as plasmids and transposons). The expression of β-lactamase is strictly regulated, involving many factors such as structural genes, repressor proteins, and antirepressor proteins. Repressor proteins negatively regulate the gene expression of β-lactamase, while antirepressor proteins are induced in the presence of β-lactamides to promote the expression of β-lactamase by relieving the inhibition of repressor proteins [18].

The beta-lactamases in MRSA can be divided into several different classes, including Group A (serine proteases), Group B (metalloenzymes), Group C (cephalosporin enzymes), and Group D (oxacillinases). The production of these enzymes can be transmitted through the bacteria’s chromosomes or plasmids, and their active sites bind to metal ions or other amino acid residues, thus participating in substrate recognition and catalytic processes. With the discovery of the New Delhi metallo-β-lactamase-1 (NDM-1) superbug in 2008, researchers have increasingly turned their attention to metal-beta-lactamase, which inactivates almost all beta-lactamides and is a key concern in the mechanism of MRSA resistance [7,28]. Studies have found that metallic β-lactamase is sensitive to amtronam and can be inhibited by EDTA, phenazoline, and sulfhydryl compounds [29], thus providing a new idea for clinical MRSA drug guidance.

MRSA’s β-lactamases are not limited to their effects on β-lactam antibiotics; they can also be extended to other types of antibiotics, such as macrolides and tetracyclines. Hashizume et al. revealed the potential reversal mechanism of β-lactam antibiotic resistance through in-depth studies [30]. In the treatment of MRSA resistant infections, the natural lipopeptide antibiotic tripeptide C not only shows significant antibacterial activity but also can effectively reduce the expression level of key resistance gene structure genes and *mecA* in MRSA. This finding suggests that tripeptide C may weaken the drug-resistant phenotype of bacteria by affecting transcriptional regulation of these genes. In addition, this resistance reversal effect was more significant when tripeptide C was combined with beta-lactam antibiotics, suggesting that tripeptide C could be used as an adjunct therapy to improve therapeutic efficacy and reduce the development of resistance in combination with conventional antibiotics.

#### 2.2.2. Modification Enzymes

The formation of aminoglycoside antibiotic resistance is closely related to the acquisition of aminoglycoside-modifying enzymes (AMEs) in bacteria. The genes encoding AMEs are usually mediated by mobile genetic elements (MGEs) such as transposons, plasmids, and integrons, which allow them to spread easily between different bacteria. AMEs reduce the binding affinity of antibiotics to bacterial ribosomes through chemical modification, thus weakening its bactericidal activity. Specifically, acetyltransferase (AAC), phosphotransferase (APH), and nucleotide transferase (ANT) are the three major enzymes that cause resistance to aminoglycoside antibiotics [31]. Among them, the AAC(6′)/APH(2′′) enzyme, which has AAC and APH activities, plays a central role in the formation of MRSA resistance to gentamicin, tobramycin, and kanamycin. This enzyme not only catalyzes acetylation and phosphorylation reactions but also accelerates the diffusion of drug-resistance genes in bacterial populations through horizontal gene transfer mediated by complex transposon *Tn4001* [32].

In addition, the ANT(4′)-I enzyme, encoded by the *ant(4′)-Ia* gene, contributes significantly to the resistance of aminoglycoside antibiotics such as neomycin, amikacin, tobramycin, and kanamycin. The *ant(4′)-Ia* gene usually carries small plasmids that are subsequently integrated into larger binding plasmids, such as pSK41, and further integrated into the SCCmec of some *Staphylococcus aureus* isolates, thus becoming stable in the bacterial genome. It is worth noting that although the role of the APH(3′)-III enzyme in MRSA resistance to neomycin and kanamycin has not been fully elucidated, it is known that its coding gene *Tn5405* can be located on chromosomes and plasmosomes, suggesting that it may play a role in the formation of resistance.

The genetic mechanism of streptomycin resistance is relatively complex, involving several factors such as the *ant(6)-Ia* gene, chromosome mutation (*strA*), *aph(3′)-III* gene, and *ant(4′)-Ia* gene [33]. In view of the importance of aminoglycoside antibiotics in clinical treatment and the urgency of drug resistance, the development of efficient inhibitors of aminoglycoside-modified enzymes has become the focus of current research. Antisense oligonucleotide analogues, as a promising drug candidate, block the translation process by specifically binding to the mRNA of the target enzyme [34], and are expected to become a new strategy to overcome aminoglycoside antibiotic resistance.

### 2.3. Drug Efflux Pump

MRSA has highly complex resistance to multiple antibiotics, partly due to its powerful efflux pump. The efflux pump, also known as the active efflux system, is a normal physiological structure of bacteria and also exists on sensitive strains. Efflux pumps are efflux proteins present on the cell membrane of *Staphylococcus aureus*. When these proteins are active, they can pump antibiotics out from the inside of the bacteria, thus reducing the concentration of antibiotics in the inside of the bacteria and making it impossible for antibiotics to effectively inhibit or kill the bacteria [9]. MRSA’s efflux pump system consists of several different pumps that recognize many antibiotic classes and other harmful substances and use energy (usually ATP) to pump these substances out of the cell to achieve resistance.

#### 2.3.1. Quorum-Sensing (QS) System-Mediated Regulation

The QS system is a system that relies on a series of small, diffusible, and specific signal molecules to regulate bacterial information exchange. When the concentration of signaling molecules reaches a certain threshold, the QS system can use them to mediate the transmission of information between bacterial species and adjust and unify each other’s behavior patterns accordingly. In this way, bacteria can jointly complete some complex physiological functions and fine regulatory mechanisms that cannot be achieved by individuals alone, such as the bioluminescence phenomenon, horizontal transfer of plasmids, production of various toxins, and induction of bacterial resistance to antibiotics [35].

MRSA’s QS system can not only directly mediate the regulation of membrane efflux pump but also participate in the formation of its biofilm. *agr* and *luxS* are the two main systems in the QS system, and the generation of known toxic factors and drug resistance in *Staphylococcus aureus* is controlled by the *agr* system [36,37]. The *agr* system regulates more than 70 genes involved in toxigenesis and drug-resistance induction. Its regulatory mechanism includes activation of *agrA*, *agrB*, and *agrC* genes to produce AIP signaling molecules, which are then excreted from cells via ABC transporters or membrane channel proteins.

The QS system indirectly promotes the development of antibiotic resistance by enhancing bacterial efflux pump activity. Buroni et al. proposed that signaling molecules produced by drug-resistant bacteria activate efflux pumps, thereby increasing the efficiency of drug extrusion. This process subsequently stimulates the release of quorum-sensing signaling molecules (e.g., AHLs), which further activate the QS system. Consequently, this cascade significantly exacerbates both drug resistance and virulence in MRSA [38].

#### 2.3.2. Functional Proteins Responsible for Membrane Transport

The effluents identified in *Staphylococcus aureus* were mainly divided into six categories: major facilitator superfamily (MFS), staphylococcal multi-drug-resistance (SMR) family, multi-drug and toxin compound extrusion (MATE) family, ATP-binding cassette (ABC) superfamily, drug metabolite transporter (SLCO) superfamily, and resistance-nodulation division (RND) family. In the process of drug efflux, superfamilies and SMR and MATE families play a leading role, and they use the energy generated by ATP hydrolysis to transport drugs. The ATP-binding box superfamily enables transmembrane transport of drugs by exploiting differences in ion concentration (such as H^+^ or Na^+^) or chemical concentration.

At present, at least 10 multi-drug pumping proteins have been found in *Staphylococcus aureus*, including NorA, NorB, NorC, MdeA, SepA, MepA, and SdrM encoded by chromosomes in the MFS family, and QacA/B and Smr encoded by plasmids in the MFS family and SMR family [39]. The action mechanism and effect of different proteins are different. Overexpression of the NorA efflux pump is closely related to the resistance of *Staphylococcus aureus* to fluoroquinolones, and it can efficiently pump such drugs out of the cell, reducing its concentration in bacterial cells and failing to achieve effective bacteriostasis [40]. Studies have found that the NorA efflux pump can not only efflux fluoroquinolone antibiotics, but also efflux Nile red, berberine, ethiobromide, acridine yellow, chlorin, etc.; so, NorA is regarded as an effective target to inhibit the MRSA efflux system [41].

In addition, MRSA resistance to macrolides and tetracycline antibiotics was also associated with the presence and expression of specific efflux pump genes. Among them, the *Msr(A)* gene-mediated ATP-binding box transporter is responsible for the active efflux of macrolide antibiotics, which reduces intracellular drug concentration by using the ion gradient, leading to drug resistance [39]. The mechanism of resistance to tetracycline drugs is similar, and the proteins encoded by the *tetK* and *tetL* genes resist drugs by pumping them out of the cell, so that they cannot reach effective antibacterial concentrations. Notably, the *tetK* gene makes *Staphylococcus aureus* resistant to tetracycline, while the *tetM* gene makes it resistant to both tetracycline and minocycline. The incidence of the *tetM/tetK* gene combination in MRSA is higher than that in MSSA, and the MIC value of MRSA isolates carrying these two genes is higher, suggesting that the *tetM* gene may play an important role in the resistance of MRSA to tetracycline antibiotics [42].

### 2.4. Formation of Biofilms

The widespread prevalence of MRSA is inseparable from the formation of biofilms, a thick, gelatinous protective layer in which bacteria can grow and reproduce. The formation of biofilms increases the resistance of MRSA to multiple antibiotics, allowing it to survive in a variety of poor survival environments, which are difficult to clear and may lead to the persistence or recurrence of the infection. A bacterial biofilm (BBF) is an adhesive structure formed by bacteria on the surface of objects consisting of fibrin, polysaccharide, lipid protein, etc. It can be composed of a single or multiple bacterial species, and is rich in biological macromolecules such as polysaccharide, peptidoglycan, protein, nucleic acid, and phospholipid [14]. The BBF not only provides physical protection for bacteria and reduces the penetration of drugs such as antibiotics but also promotes horizontal gene transfer and increases the resistance of bacteria. In addition, the microenvironment generated by bacterial metabolic activity within the biofilm may also affect the effectiveness of the drug. Therefore, the in-depth study of the MRSA biofilm formation mechanism, structural characteristics, and its influence on drug sensitivity is of great significance for the development of new antimicrobial strategies and therapeutic methods.

#### 2.4.1. Formation of MRSA Biofilm and Regulation of Related Genes

The formation of MRSA biofilms usually follows a certain sequence: (i) Bacterial adhesion stage. At this time, the free bacterial cells recognize and adhere to the surface of the host cell or the surface of an inanimate object such as a medical device through a specific molecular mechanism; (ii) Aggregation and proliferation stage of bacteria. After adhesion, the bacteria begin to adhere to each other, form multicellular structures, and proliferate rapidly under suitable environmental conditions; (iii) Maturity stage of biofilm. With the increase in the number of cells and the accumulation of extracellular matrix, the biofilm gradually becomes more stable and complex; and (iv) Bacterial shedding and dissemination stage. Bacteria in mature biofilms may escape the biofilm through some mechanism and enter the surrounding environment, thereby triggering new infections [10].

At the molecular level, the formation of MRSA biofilms involves a series of key molecular and gene interactions. Autolysin A is an important factor that helps bacteria adhere to the surface of host cells. Another key factor is polysaccharide intercellular adhesin (PIA), which is encoded by the *ica* locus and is conducive to bacterial intercellular adhesion and biofilm formation [43]. *ica* loci include genes such as *icaR* and *icaADBC*, which together regulate PIA synthesis. Among them, the *icaA* gene plays a decisive role in biofilm formation [44]. Some researchers used RT-PCR technology to find that under the action of magnolol, the expression level of the *icaA* gene in MRSA decreased significantly, resulting in a strong inhibition of biofilm formation [45]. Since the four homologous proteins of *icaABCD* are controlled by the same operon, the transcriptional activity of *icaA* directly reflects the activity state of the whole operon. In addition, *ica* gene expression is also regulated by other genes such as *SarA*, *SigB*, *Agr*, etc. These genes indirectly control biofilm generation by influencing PIA synthesis.

In addition, there are a variety of adhesion proteins in the extracellular matrix of biofilm, such as Bap, Aap, SasG, SasC, Embp, etc. These proteins can regulate the formation of biofilm by the *ica* independent pathway. In recent years, extracellular DNA (eDNA) has received increasing attention in biofilm research, which not only helps maintain the stability of biofilm, but also may participate in gene transfer between bacteria [46]. The ability of MRSA strains to form biofilms varied from strain to strain, which was related to the Spa genotype carried by the strain, but had relatively little relationship with the *MLST* gene and *SCCmec* gene.

#### 2.4.2. Resistance Mechanisms Mediated by MRSA Biofilm

The resistance of MRSA is closely related to its biofilm formation ability, involving multiple mechanisms such as nutritional restriction, osmotic barrier, and quorum sensing [47]. The formation mechanism of the biofilm is complex, mainly including providing protection for bacteria, resisting the attack of antibiotics and the immune system, and mediating the damage of immune cells through the matrix barrier and quorum sensing. In the biofilm of MRSA, the bacteria form a sticky protective layer by secreting extracellular polymeric substances (EPS), which not only helps the bacterial cells adhere to each other but also resists the penetration of the host’s immune cells and antibiotics. In addition, the microenvironment inside the biofilm, such as low oxygen and local concentration changes of nutrients, provides a special environment conducive to the survival of bacteria [48]. In this environment, bacteria can adjust their metabolic pathways to adapt to limited resource conditions.

De kievit et al. pointed out that bacteria in biofilms can also communicate through the QS system, which allows bacteria to sense the density of similar bacteria around them and initiate a specific gene expression program when a certain threshold is reached [49]. These procedures may involve the maintenance of biofilms, the expression of resistance genes, and the production of virulence factors. Through quorum sensing, bacteria within the biofilm are able to coordinate behavior, such as producing biofilm enzymes to destroy host tissue, or releasing free bacteria when necessary to find new sites of infection.

When MRSA forms a biofilm, its direct contact with host tissue is reduced, reducing the inflammatory response and allowing the bacteria to survive in the body and maintain a chronic infection. Although antibiotics are effective against free bacterial cells, the barrier function of the biofilm and the obstruction of the body’s defense system make it difficult for antibiotics to reach and kill the bacteria in the membrane, and the biofilm can also continue to release new free bacteria, making it difficult to completely cure the infection [10]. It can be seen that by studying the formation mechanism of bacterial biofilm, going deep into the gene and molecular level, and intervening in its formation process through relevant technical means, the understanding of bacterial infection will be more thorough, so as to carry out more effective prevention and treatment, and provide a new research direction for further development of new drugs and reversal of bacterial resistance.

### 2.5. The Change of Antibacterial Targets and Affinity

#### 2.5.1. Changes in the Targets of Antibacterial Agents

##### Resistance Mechanisms of MRSA to Vancomycin

Vancomycin and teicoranin are the main glycopeptide antibiotics used in the clinical treatment of MRSA infection, and their mechanism of action is to block the synthesis of bacterial cell walls by interacting with key steps in cell wall mucopeptide formation [50]. Vancomycin has been widely used in clinical practice because of its remarkable efficacy. However, with the increase in the frequency of use, some MRSA strains with reduced susceptibility to vancomycin (such as vancomycin-intermediate sensitive *Staphylococcus aureus*, VISA) and vancomycin-resistant MRSA strains (such as vancomycin-resistant *Staphylococcus aureus*, VRSA) appear, posing new challenges to MRSA treatment.

Vancomycin resistance mechanisms are complex and diverse, the most important of which is the synthesis of D-alanine-D-lactic acid, the low-affinity peptidoglycan precursor encoded by *van* operon in plasmid Tn1546, which leads to vancomycin’s inability to effectively bind to its target site, resulting in drug resistance [51]. Lipid II in strain VISA contains cell wall peptidoglycan structural units terminated by D-alanine-D-lactic acid residues. These termination units may act as the wrong target of vancomycin, capture part of the drug, and reduce the binding of vancomycin to the true target, resulting in reduced sensitivity. In addition, the possible gene mutations in the VISA strain, such as *walkR*, *vraSR*, and *rpoB* genes, as well as the up-regulated expression of the cell envelope response genes *vraSR* and *mprF*, may adapt to the antibacterial action of vancomycin by changing the cell envelope characteristics of the bacteria, thus reducing its sensitivity [52]. Studies have shown that genetic regulatory factors such as the *agr* gene in *Staphylococcus aureus* are also closely related to vancomycin sensitivity [53].

Although there are fewer cases of vancomycin treatment failure in the current clinic, in the case of VISA or VRSA strains, doctors can consider using alternative drugs such as dattomycin, linezolid, ceflorin, etc., or select the appropriate drug based on the results of drug susceptibility tests. In addition, the combination of beta-lactam drugs and vancomycin has been shown to have a synergistic effect on MRSA [54], which provides additional treatment options for clinical treatment. In conclusion, understanding the mechanisms of resistance to MRSA and monitoring the prevalence of resistant strains are essential to guide the rational use of antibiotics and develop effective treatment strategies.

##### Resistance Mechanisms of MRSA to Linezolid

Linezolid, as a first-line drug for the treatment of MRSA infection, has shown remarkable efficacy in clinical practice. However, in recent years, with the widespread application of linezolid, the resistance of MRSA to linezolid has gradually become prominent, mainly due to the structural changes and gene mutations of bacterial ribosomes [55].

Point mutation of bacterial ribosomal 23S rRNA is one of the main mechanisms of linezolid resistance. This mutation alters the target of linezolid, making it impossible for the drug to effectively bind to the ribosome, thereby inhibiting protein synthesis in the bacteria. Notably, this point mutation is a genetic mutation that develops spontaneously, as a result of long-term use of linezolid, and this mutation cannot be transferred between bacteria, which means that once resistance develops, other means are needed to control its spread. Among all point mutations, the G2576T mutation is the most common mutation site [56].

In addition to point mutations in ribosomal 23S rRNA, amino acid mutations in ribosomal L3 or L4 proteins are also important factors leading to linezolid resistance. These mutations cause changes in the structure of the ribosome that prevent linezolid from binding effectively to the ribosome. In addition, the methyltransferase gene *cfr* encodes RNA methylase, which causes most ribosomes to be methylated at A2503 of 23S rRNA, making MRSA resistant to ribosome-targeted linezolid [57]. The plasmid pSCFS7, as a carrier of this gene, can be efficiently transferred among different MRSA strains, which further exacerbates the spread of resistance. In view of the serious situation of resistance to linezolid, the scope of its indication should be strictly controlled in clinics to avoid unnecessary overuse.

##### MRSA Resistance to Other Drugs Caused by Changes of Action Targets

Macrolide resistance: The mechanism of resistance of macrolide antibiotics to MRSA mainly involves methylation of specific adenine residues of 23S rRNA, a process catalyzed by methyltransferase encoded by the *erm* gene. The main subtypes of the *erm* gene include *ermA*, *ermB*, *ermC*, and *ermF*, among which *ermA* and *ermC* are more common in clinically isolated MRSA [58]. The activity of these methyltransferases results in the inability of macrolide antibiotics to effectively bind to their target sites in bacterial ribosomes, thereby diminishing the antimicrobial activity of the drugs. In addition, plasmid-mediated horizontal gene transfer further promotes the spread of *erm* genes [59,60], exacerbating the problem of drug resistance.

Quinolone resistance: Quinolone antibiotics have good antibacterial activity against MRSA by inhibiting bacterial DNA rotase and topoisomerase IV, blocking DNA replication and transcription processes. However, the main mechanism for the development of resistance is point mutations in bacterial DNA rotation enzymes (such as the *gyrA* and *gyrB* genes) and topoisomerase IV (such as the *parC* and *parE* genes) [61], which reduce the affinity of quinolones to their targets, leading to drug failure. In addition, bacteria may also enhance resistance to quinolone antibiotics by increasing the expression of drug efflux pumps, such as overexpression of the *norA* gene [62].

Tetracycline resistance: Tetracycline antibiotics inhibit protein synthesis by inhibiting the function of 30S subunits of bacterial ribosomes and blocking the correct pairing of aminoacyl-tRNA on the mRNA template. Tetracycline resistance is often associated with genes in bacteria that code for ribosome protection proteins that are able to bind to tetracycline, preventing its binding to ribosomes and thus making bacteria resistant to tetracycline [63,64].

#### 2.5.2. Changes of Target Affinity of Antimicrobial Agents

The resistance mechanism of MRSA to antibiotic and target affinity changes is mainly reflected in the resistance to cephalotrin. Cefalotrin, a fifth-generation cephalosporin antibiotic, was approved by the Food and Drug Administration (FDA) in 2010 for the treatment of Complicated Skin and Soft Tissue Infections (cSSSIs) and Community-Acquired Pneumonia (CAP). The remarkable antibacterial activity of ceflorin, especially against MRSA, is attributed to its high affinity for PBP2a [65]. However, in recent years, MRSA resistance to cephalotrin has increased, a phenomenon that has prompted clinical practice to re-evaluate its use in the treatment of MRSA infections.

In some MRSA strains, the specific amino acid site of the *mecA* gene mutated, leading to structural changes in PBP2a, which weakened the interaction between ceflorin and PBP2a, making the drug unable to effectively inhibit bacterial cell wall synthesis. By studying MRSA isolates from Nigeria [66], a triple mutation pattern of the *mecA* gene (N146K-N204K-G246E) was found to be associated with increased resistance to ceflorin. This finding provides molecular evidence for understanding the mechanism of ceflorin resistance. In addition, Wüthrich et al. also pointed out that *N204K* mutation in *mecA* gene is related to MRSA resistance to cephalotrin [67]. In summary, the problem of resistance of ceflorin as an effective anti-MRSA drug has attracted widespread attention, which also highlights the critical role of rational antibiotic use and effective infection control measures in slowing the development of resistance.

## 3. Novel Treatment Strategies for MRSA

As an epidemic, rapidly mutating, multi-drug-resistant bacteria, MRSA’s widespread spread and the continuous emergence of drug-resistant strains pose a serious threat to public health, and it is urgent for us to find effective suppression means. At present, new antibiotics such as linezolid [68] and semi-synthetic drugs such as tigacycline have been used to treat multi-drug-resistant *Staphylococcus aureus* infection, and the efficacy is still good in clinical practice. In addition, antibiotic combination strategies, such as the combination of fusedic acid with doxycycline, cefquinoxime with enoxacin, etc. [69], have also been proven to significantly enhance the antibacterial effect [70,71] (Table 1). However, it is difficult to completely solve the problem of bacterial resistance with existing antibiotics alone, and new drug use strategies and novel treatment methods have yet to be explored to address this growing challenge.

### 3.1. Combined Therapy

At the 34th annual congress of the European Society of Clinical Microbiology and Infectious Diseases (ESCMID Global 2024), the CAMERA-2 research team presented their findings on the treatment of MRSA bacteremia. In one study, vancomycin or daptomycin combined with anti-staphylococcal beta-lactam drugs showed some synergistic effect in the treatment of MRSA bacteremia [72]. The results showed that when in vitro combination susceptibility tests suggested a positive interaction between vancomycin/dattomycin combined anti-staphylococcal beta-lactam drugs, this antimicrobial combination was associated with a lower 14-day all-cause mortality.

In addition, combining antibiotics with different mechanisms can also improve treatment effectiveness and reduce the development of resistance. For example, the combination of cefquinme with enrofloxacin, Genamicin, and kanamycin, as well as the combination of vancomycin with levofloxacin, rifampicin, fosfomycin, and imipenem, have been proven to effectively inhibit the growth of MRSA [73]. Additionally, a previous study demonstrates that the cefepime-imipenem/cilastatin combination exerts synergistic bactericidal effects against MRSA, reducing colony counts by 3–4 log_10_ CFU/mL within 24 h while significantly lowering the MIC_90_ at clinically achievable concentrations [74]. Mechanistically, this regimen restores MRSA susceptibility to cephalosporins through cross-sensitization, thereby delaying the emergence of resistance mutations. Separately, drug synergy screening has identified multiple β-lactam antibiotics that enhance ceftobiprole’s antibacterial efficacy when used in combination against MRSA strains [75].

### 3.2. Bacteriophage Therapy

Bacteriophages are a special class of viruses that are specialized in infecting and destroying bacteria, which can be divided into lytic and lytic prototypes. The principle of phage therapy is to use natural enemies to fight bacterial infections, and it has great potential for treating antibiotic-resistant bacterial infections. Among many bacteriophages, bacteriolytic bacteriophages have greater application prospects in medicine because they can directly cause bacterial rupture. Phages have the ability to specifically target and destroy certain bacterial strains, including those that form biofilms. Phages have developed different mechanisms for penetrating and destroying biofilms, with one important strategy involving the production of depolymerase [76]. Phages can produce depolymerase enzymes that potentially destroy the extracellular matrix of the biofilm, breaking down the EPS present in it. In the context of phage therapy and biofilm eradication, research has made progress in identifying various phage species that show potential against MRSA and can be used as a complementary or alternative therapy to antibiotics [77].

In the study of phage therapy for MRSA, scientists have isolated and identified a series of specific phages, such as MR-10, MR-11, MR-12, and MR-14, which have shown significant inhibition of MRSA in laboratory tests and animal models [78,79,80]. They are able to specifically recognize and destroy MRSA cells, thereby reducing the number of bacteria. Meanwhile, in a study published in the journal *Antimicrobial Agents and Chemotherapy*, researchers evaluated the efficacy of a cocktail of multiple phages against MRSA biofilms [81], which contain phages capable of targeting different strains of MRSA. The results showed that the phage cocktail was able to effectively reduce the biomass of the biofilm, destroy its structure, and increase the susceptibility of MRSA to antibiotics, thus promoting the removal of bacteria. This suggests that phage cocktails may be an effective drug for the treatment of clinical biofilm infections [82].

In addition, positive therapeutic results using customized phage cocktails in clinical case studies have further stimulated the medical community’s interest in phage therapy. Several clinical trials are currently underway worldwide to verify the efficacy and safety of phage therapy for MRSA.

### 3.3. Nanobiologic Therapy

Nanobiotics are a special class of nanomaterials that possess antimicrobial properties or can enhance the efficacy of existing antibiotics [83]. These materials have a wide range of applications in the medical field, for example, they can be used as surface coatings on implantable medical devices to prevent bacterial infections [84]. The use of nanobiologics against antimicrobial resistance (AMR) pathogens such as multi-drug-resistant bacteria (such as MRSA) represents an innovative therapeutic strategy and highlights the current challenges in the fight against AMR bacteria [85].

Nanobiotics exert their antibacterial effects through various mechanisms. Because the size of the nanoparticles (NPs) allows them to act directly on individual bacterial cells, they can effectively increase the effectiveness of antibacterial drugs and potentially reduce the risk of bacteria developing resistance. In addition, some nanoparticles themselves have antibiotic properties. Currently, several nanoparticles have been studied for the treatment of bacterial infections, including silver nanoparticles (silver NPs), zinc oxide nanoparticles (ZnO NPs), and gold nanoparticles (Au NPs). Among them, silver nanoparticles (Ag NPs) have become one of the most eye-catching nanobiologic agents due to their excellent antibacterial activity [86,87].

Silver nanoparticles are tiny silver particles created using nanotechnology, ranging in diameter from 1 to 100 nanometers. Due to their nanoscale size, silver nanoparticles have an extremely high surface-area-to-volume ratio, which significantly enhances their broad antimicrobial properties [88]. Silver nanoparticles have been widely used in many fields because of their unique magnetic, optical, and electrical properties and strong antibacterial ability. The reason why silver nanoparticles are favored is mainly due to their multiple killing mechanisms against bacteria. They can not only directly adsorb on the cell wall of bacteria, destroying the integrity of its membrane structure, but also penetrate the interior of cells, causing more cell damage until the bacteria lose their basic life activities. In addition, silver nanoparticles can also produce free radicals and reactive oxygen species, which carry out oxidative attacks on bacteria. At the same time, they can interfere with key signaling pathways required for bacterial replication, making them a potential alternative therapy for the treatment of MRSA infections [89,90,91]. Taken together, these diverse and powerful properties of silver nanoparticles and other related substances make them ideal candidates to fight MRSA infection.

### 3.4. Antimicrobial Peptides (AMPs)

AMPs are an integral part of the host immune system. AMPs are usually composed of 10 to 50 amino acid residues, which are positively charged short chain amino acids that give them amphiphilic properties, allowing them to easily penetrate the bacterial cell membrane, damage its structure, and cause the cell contents to leak, resulting in bacterial death [92]. Studies have shown that AMPs have a significant bactericidal effect on resistant bacteria such as MRSA. AMPs disturb the lipid bilayer by interacting with the negative charge on the bacterial cell membrane, leading to instability and destruction of the membrane structure [93]. Once AMPs are inserted into the bacterial cell membrane, they interfere with ion balance, creating pores or channels that allow calcium and potassium plasma to flow into the bacterial cell, and this abnormal flow of ions disrupts the basic functions of the bacteria, ultimately leading to cell death [94]. AMPs not only have an effect on cell membranes, but also interfere with protein folding in bacteria, destroy cell walls, and inhibit enzyme activity. AMPs have received a lot of attention as a potential alternative treatment option for multi-drug-resistant organisms (MDROs) [95].

AMPs may also increase the permeability of bacterial cell membranes, leading to the leakage of important metabolites and essential substances such as nucleotides, amino acids, and ATP in cells, further weakening the viability of bacteria [96]. Some AMPs also have the ability to penetrate bacterial cell membranes and target intracellular components, and they can interact with intracellular proteins, RNA, or DNA to disrupt bacterial growth and replication mechanisms [97]. In addition, AMPs can affect the host’s immune system while directly fighting microorganisms. They are able to stimulate the release of immune mediators such as chemokines and cytokines, promote the aggregation of immune cells to the site of infection, and trigger an inflammatory response, helping to eliminate infection and strengthen the host’s defense against MRSA [98].

Currently, AMPs have been used to treat wound infections caused by MRSA. AMPs promote wound healing by topical application to the wound site, such as dressing, ointment, gel, or cream, acting directly on the infected area [99]. In addition, AMPs also exhibit immunomodulatory properties [100]. Moreover, when used in combination with standard antibiotics, AMPs can enhance the effectiveness of the treatment of MRSA infections, helping to overcome resistance through synergies. This combination therapy can not only help eradicate MRSA bacteria and slow the spread of new infections, but also speed up the healing process of wounds [101]. In addition to this, researchers are working to develop bioengineered AMPs with improved stability and enhanced antimicrobial activity. Bioengineering technology can improve the stability of AMPs, reduce their toxicity, and enhance their specific targeting ability against MRSA and other resistant bacteria [102].

### 3.5. Live Bio-Therapeutics

The use of beneficial microbes for treatment, so-called “live biotherapy,” which introduces non-pathogenic strains to compete with pathogenic bacteria for living space and assist in the removal of these harmful bacteria, has become an emerging therapeutic approach. With the deepening understanding of the function of the human microbiome, the use of probiotics as supplements has become increasingly popular as a key element in regulating and restoring microbial balance [103]. Probiotics are a class of bacteria that are crucial to host health, and they establish a symbiotic relationship with the host, and moderate intake of probiotics is beneficial to human body [104]. The main mechanisms of action of probiotics include enhancing the epithelial barrier, promoting the adhesion of intestinal mucosa, inhibiting the attachment and growth of harmful microorganisms through competitive rejection mechanisms, producing antimicrobial substances, and regulating the host immune response [105,106,107]. During antibiotic treatment, the microbiota can be disrupted, which provides an opportunity for the selection and spread of resistant strains of bacteria such as MRSA. Evidence confirms that probiotics enhance antibiotic therapeutic efficacy by modulating gut microbiota composition and reducing populations of antibiotic resistance gene-carrying bacteria [108].

Certain probiotic strains have been shown to be effective in inhibiting the growth and adherence of resistant pathogens such as MRSA [109], suggesting the potential of probiotics in preventing and reducing the formation of antibiotic resistance. Probiotics play a therapeutic role by maintaining a healthy balance of the microbiome, reducing dependence on antibiotics, and through a variety of mechanisms, including immune regulation, regulation of the gut microbiota, production of antimicrobial substances, and competitive rejection [110]. In addition, probiotics play a key role in fighting infection by blocking and disrupting pathogen biofilm formation by inhibiting pathogen binding to host cell receptors, producing antimicrobial substances, improving host immune surveillance, and eliciting appropriate inflammatory responses [111].

Common probiotics include *Lactobacillus*, *Enterococcus*, *Bacillus*, *Streptomyces*, *Saccharomyces cerevisiae*, *Corynebacterium honor*, and Nisin derived from *Lactobacillus*. Numerous studies have shown a synergistic effect of probiotics and antimicrobial-based therapies in the treatment of bacterial and fungal infections [112]. The biofilm-forming ability of *Staphylococcus aureus* is an important factor in its antibiotic resistance, which can lead to severe and difficult-to-cure MRSA infections. Probiotics can effectively prevent the formation of pathogen biofilms and play a role in nutrient competition, thus playing an important role in the fight against infection. In addition, the use of probiotics can also significantly reduce AMR genes carried by the hospital surface microbiota. One study showed that adopting probiotic-based hospital hygiene was able to reduce the frequency of AMR genes carried by the hospital surface microbiota by up to 99% [113].

### 3.6. Chinese Herbal Drugs Therapy

A number of studies have found that the extracts of Chinese medicinal materials such as *gallgall*, *Coptis chinensis*, *Phellodendron phellodendron*, *Scutellaria scutellaria*, *knotweed*, *rhubarb*, *liquorice*, *bupleurum*, *gentian hosta*, and *Jiuliming white flower* have significant antibacterial and even bactericidal effects on MRSA [114]. For example, garlic oil has shown strong bactericidal activity against MRSA strains, and the bactericidal mechanism is related to its interference with protein synthesis [115].

Despite this, not all combined applications of Chinese herbs enhance anti-MRSA activity. Ren et al. showed that the compatibility of gallnut and scutellaria significantly improved the bacteriostatic effect on MRSA, showing a synergistic effect. However, the antibacterial effect of the combination of *Coptis chinensis* and scutellaria chinensis was not as good as that of *Coptis chinensis* alone, showing antagonistic effects. This may be because the alkaloids in Coptis coptis and the flavonoids in scutellaria scutellaria precipitated during the co-decoction process, which weakened the antibacterial activity [116]. In addition, Ren et al. also found that the compatibility of rhubarb and matrine had an additive effect on MRSA, while the compatibility of rhubarb and Coptis showed an antagonistic effect. At the same time, the antibacterial effect of Chinese herbal extracts obtained by different extraction methods, such as alcohol extract and water extract, may be significantly different, and the antibacterial activity of Chinese herbal extracts will also vary with the change in concentration [117].

In addition, scholars have found that certain Chinese herbal extracts can work synergistically with antibiotics and even help reverse bacterial resistance to certain antibiotics [118]. Cui et al. used ethanol and water as extractants to extract active components from eucalyptus leaves, and observed their antibacterial effects on MRSA strains, finding that the eucalyptus extract combined with oxacillin had significant synergistic antibacterial effects on MRSA strains [119]. Wu et al. found through experiments that when the extract of cassia alcohol is combined with oxacillin, it has an inhibitory effect on the biofilm formation of MRSA, thus playing a synergistic bactericidal role [120]. In addition, studies showed that cassia could reduce the expression of some toxins in MRSA strains [121]. A study conducted resistance reversal tests on 14 kinds of Chinese medicine drug-containing serum, such as gallnut, parsnips, scutellaria, etc., co-cultured with MRSA, and found that serum containing these herbs could restore MRSA’s sensitivity to β-lactam antibiotics [122]. To sum up, Chinese herbal medicine has a wide range of application prospects in anti-MRSA treatment, but it is necessary to scientifically and reasonably select the extraction method, ensure that the extract reaches an effective antibacterial concentration, and consider the interaction between drugs to achieve the best therapeutic effect.

## 4. Conclusions and Future Perspectives

In epidemiology, MRSA has the characteristics of a wide spread, strong infectivity, high fatality rate, and high fatality rate. The resistance of MRSA varies from location to location. Due to its insensitivity to multiple antibiotics, MRSA has become a global public health problem. In this paper, the drug-resistance mechanism of MRSA mainly includes inherent resistance mechanism, acquired resistance mechanism, biofilm formation, and the change in antimicrobial targets and their affinity. These mechanisms enable MRSA to evade the killing effect of antibiotics, forming a complex multi-drug-resistance mechanism, which increases the difficulty of treatment. At the same time, this paper also reviewed the latest treatment strategies on MRSA, and briefly expounded the application of combination therapy, phage, AMPs, and other therapeutic measures. Among them, the research and development of new drugs for MRSA is the key. The emergence of new antibiotics such as linezolid and tigecycline provide a new choice for the treatment of MRSA. These drugs inhibit bacterial growth through different mechanisms and have good clinical effects, which brings new hope for the treatment of MRSA.

Today, we face the twin challenges of growing resistance and lagging development of new antibiotics. In order to effectively deal with this situation, we need to take a multi-faceted approach. First, strengthen infection control measures to reduce the spread of MRSA. Secondly, optimize antibiotic use strategies to avoid unnecessary antibiotic use and reduce the generation of drug resistance. Thirdly, basic scientific research should be strengthened to deeply understand the resistance mechanism of MRSA and provide theoretical support for the research and development of new antibiotics. Finally, strengthen international cooperation to jointly tackle the global problem of drug-resistant bacteria. Going forward, we look forward to continued research efforts that will facilitate the discovery of new antibiotics and therapeutic strategies to more effectively treat MRSA infections. At the same time, scientists also need to strengthen cooperation on a global scale to develop and implement effective control policies for drug-resistant bacteria to protect human health.

## Figures and Tables

**Table 1:** Summary of novel treatment strategies for MRSA.

Strategies	Substantive Contents	Action Mechanisms Against MRSA	Precautions
Combined therapy	The concurrent use of two or more antibiotics to enhance therapeutic efficacy and reduce the emergence of drug resistance.	The synergistic action among different antibiotics can more effectively kill MRSA while reducing the chances of bacteria developing resistance.	When using antibiotics with different action mechanisms in combination, it is essential to carefully assess the potential drug interactions to avoid reduced efficacy or increased toxicity.
Bacteriophage therapy	Utilizing specific viruses (bacteriophages) to infect and kill bacteria.	Phages are able to specifically recognize and infect MRSA and cause bacterial death by releasing enzymes and other factors that destroy bacterial cell walls.	The safety of bacteriophages needs to be rigorously assessed to ensure they do not adversely affect the host microbiota or elicit immune responses.
Nanobiologic therapy	Involving the use of drugs or materials prepared using nanotechnology to accurately target and treat infections.	NPs are designed to specifically recognize MRSA and treat infections by releasing drugs or directly destroying bacterial cells.	Nanoparticles, when used as drug carriers, require careful design of their size, surface properties, and release kinetics to optimize therapeutic efficacy.
AMPs	A class of naturally occurring peptide molecules with broad-spectrum antimicrobial activity.	By disrupting the membrane integrity of MRSA, AMPs leads to intracellular material leakage and bacterial death	AMPs have poor stability in the body and are easily degraded by proteases, thus requiring chemical modifications to enhance their stability.
Live bio-therapeutics	Using live microorganisms (such as probiotics) or their metabolic products to treat diseases caused by MRSA.	Controlling infections by competing for nutrients or producing substances that inhibit the growth of MRSA.	Treatment with live bacteria or their derivatives may cause immune responses and therefore needs to be monitored and managed.
Chinese herbal drugs therapy	Using natural plant extracts or other natural components to treat diseases.	The active components in traditional Chinese medicine may affect MRSA through multiple pathways, such as inhibiting its growth, disrupting its biofilms, or enhancing the host immune response.	The composition of traditional Chinese medicinal herbs is complex, necessitating the assurance of consistency in the source, extraction processes, and quality standards of the herbs.

## Data Availability

No new data were created or analyzed in this study. Data sharing is not applicable to this article.
